# A survey of sequence-to-graph mapping algorithms in the pangenome era

**DOI:** 10.1186/s13059-025-03606-6

**Published:** 2025-05-22

**Authors:** Yingbo Cui, Chenchen Peng, Zeyu Xia, Canqun Yang, Yifei Guo

**Affiliations:** 1 College of Computer Science and Technology, National University of Defense Technology, No.137 Yanwachi St, 410073 Changsha, People’s Republic of China; 2 National Supercomputer Center in Tianjin, No.10 Xinhuan West Rd, 300457 Tianjin, People’s Republic of China

**Keywords:** Pangenome, Sequence-to-graph Mapping, Seed-and-extend

## Abstract

A pangenome can reveal the genetic diversity across different individuals simultaneously. It offers a more comprehensive reference for genome analysis compared to a single linear genome that may introduce allele bias. Pangenomes are often represented as genome graphs, making sequence-to-graph mapping a fundamental task for pangenome construction and analysis. Numerous sequence-to-graph mapping algorithms have been developed over the past few years. Here, we provide a review of the advancements in sequence-to-graph mapping algorithms in the pangenome era. We also discuss the challenges and opportunities that arise in the context of pangenome graphs.

## Introduction

In modern genomics, reference genomes are the baselines of many genomic analyses, such as read mapping, variation detection, and phylogenetic tree analysis [1]. The current reference genomes, such as GRCh38 [2] and T2 T-CHM13 [3], are largely based on a few individuals or a single person, which may introduce reference bias [4]. Reference bias is a phenomenon that non-reference alleles in reads are underrepresented or mismapped when aligned to a linear reference genome [5–8]. Such bias may pose challenges for downstream analyses, as evidenced by a study [9] that found it led to the omission of nearly two-thirds of known structure variations. To overcome these limitations, many studies have focused on understanding and minimizing the impact of reference bias [7, 8, 10, 11]. Recently, researchers developed pangenomes, which integrates multiple linear genomes to simultaneously represent different haplotypes [12].

A pangenome is commonly represented as a graph [13, 14], where nodes represent sequences and edges represent adjacencies between sequences. In a pangenome, shared sequences across different individuals are merged into the same nodes, while individual-specific variations appear as branches. By incorporating global genomic diversity, pangenome graphs offer a more accurate and complete reference genome [1, 9, 15–17]. Current pangenome studies are typically conducted within a single species [6, 16], while there also exist studies using pangenome across species, such as great apes [18], *M. tuberculosis *strains [18], and *Solanum* species [19].

Sequence-to-graph (S2G) mapping is the process of mapping a sequence to a reference represented as a graph, which identifies the most probable path for the sequence within the graph. It is the computing core of many pangenome-related analyses, such as pangenome graph construction, variant calling, genotyping, long-read error correction, and so on [20]. In recent years, over twenty S2G mapping algorithms have been developed, with the earliest one dating back to 2009 [21]. These algorithms have collectively garnered over 10,000 citations. While several reviews cover topics such as pangenomes and graph construction [4, 6, 12, 14, 22, 23], none has provided a comprehensive review specifically focused on sequence-to-graph mapping. To our knowledge, this article is the first to provide a review of sequence-to-graph mapping algorithms. In this paper, we systematically reviewed these algorithms and summarized their characteristics to guide the application and the further research in this field.

The remainder of this paper is organized as follows: the “Pangenome graph” section introduces the concept of pangenome graphs and their common forms. The “Overview of sequence-to-graph mapping” section provides an overview of the workflow for S2G mapping. The “Seeding,” “Filtering,” and “Extension” sections review existing research on the three steps of S2G mapping algorithms: seeding, filtering, and extending, respectively. The “Other methods” section discusses mapping algorithms that use methods other than seeding and extending strategies. Finally, the “Future directions” section explores future directions of S2G mapping algorithms.

## Pangenome graph

Graphs are the most widely used pangenome representation because of their versatility and ability to capture complex genome structures [14]. Several kinds of graphs exist for representing pangenomes, each with its own unique features. A pangenome graph is generally composed of nodes and edges, with variations in how nodes are connected and the type of information each node represents. Additionally, many graph types include paths, which represent the haplotype sequences of individual genomes in the graph. Here, we introduce these graphs and their properties.

### De Bruijn graph

**Fig. 1:**
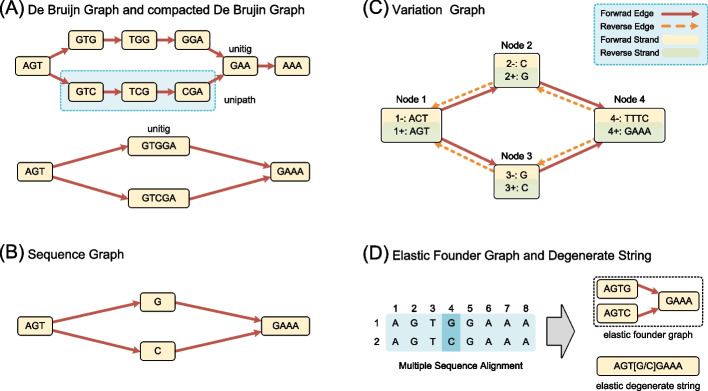
Common representations of pangenome graphs. The sequences corresponding to the four diagrams are AGTGGAAA and AGTCGAAA. **a** A De Bruijn graph, the upper part showing the $k=3$ De Bruijn graph, and the lower part depicting the compressed representation. **b** A sequence graph. **c** A variation graph. **d** A pangenome using elastic representation, which includes both the elastic founder graph and the elastic founder string forms

*De Bruijn graph* (DBG) was first introduced for genome assembly in bioinformatics [24–26]. In a De Bruijn graph, nodes are represented by fixed-length *k*-mers, and edges indicate the overlap between adjacent *k*-mers (Fig. 1a). Non-branching paths are called *unipaths*, and nodes in *unipaths* are referred as *unitigs* [27]. To reduce the size of the graphs, compacted De Bruijn graphs (cDBG) merge unitigs and create equivalent graphs [28–30], although the unitigs do not have a fixed length. Another variation of DBGs is colored DBG, with different colors representing different haplotypes [26].

### Sequence graph

A sequence graph is a directed graph in which nodes represent sequences and edges represent connections between these sequences [9]. It is often organized as a directed acyclic graph (DAG), as shown in Fig. 1b, which has only one different base, creating a branch at that point. Some sequence graphs also allow loops to model repetitive regions, such as tandem repeats [31].

### Variation graph

A variation graph (Fig. 1c) is a specialized bidirectional sequence graph with embedded paths [32]. Each node contains two strands, representing a sequence and its reverse complement. Edges are identified as the ordered pairs of oriented nodes that they link. They can be traversed in either the forward or reverse direction. For example, the edges connect reverse strands during reverse traversal. Variation graph provides path to represent the relations between the graph and other sequences [32]. A path is the sequential node mappings where each segment is aligned to a specific node. A path becomes fully embedded when all segments exactly match the corresponding nodes, which enables the representation of haplotype or reference sequence within a graph.

### Elastic representation

The methods discussed in this section, elastic founder graphs (EFG) and elastic degenerate string (ED-string), both utilize elastic representation to manage genomic data in graph structures. Although these two approaches differ in their specific implementations-EFG focusing on organizing multiple sequence alignments (MSAs) as a graph, while ED-strings simplify graph representations of small genome variants-they share the common goal of efficiently handling genomic variations. By leveraging elastic representation, both methods provide innovative solutions for indexing and pattern matching in genomic graphs.

#### Elastic founder graphs

EFGs (Fig. 1d) organize multiple sequence alignments (MSAs) as a graph [33, 34]. It splits MSAs into blocks, merges identical nodes within the same blocks, and connects the remaining nodes to form a graph. EFG has achieved a breakthrough in indexing labeled graphs by allowing for a polynomial-space index for linear-time pattern matching, surpassing the previously established lower bounds [35].

#### Elastic degenerate string

ED-string (Fig. 1d) simplifies a graph by representing small genome variants, such as single nucleotide variants and indels, as non-deterministic strings [36–38]. These ED-strings serve as nodes. Complex variants are still represented by branches of graphs [39].

## Overview of sequence-to-graph mapping

We consider “mapping” to be a more general concept than “alignment.” “Mapping” refers to the process of locating the approximate position of a sequence within a reference without precise “alignment” [6, 40]. It can also encompass the entire process, including exact “alignment,” such as GenomeMapper [21] or VG map [41]. In contrast, “alignment” specifically denotes the process of achieving base-level precision in matching, as seen in algorithms like Smith-Waterman (SW) [42] and WFA [43, 44]. Sequence alignment aims to find the best matching pattern $A^*$ with maximum similarity between two sequences, $S={s_1 s_2 \cdots s_n}$ and $T={t_1 t_2 \cdots t_m}$. The similarity of matching pattern *A* is usually determined by a score function *f*(*S*, *T*, *A*), which takes into account matches, mismatches, and gaps at each position. So, alignment is an optimization problem, as shown in Eq. (1),

1 $$\begin{aligned} A^* = \arg \underset{A \in \mathcal {A}}{\max } \left( f(S, T, A) \right) \end{aligned}$$

where $\mathcal {A}$ represents all possible matching patterns between the sequences.

**Fig. 2:**
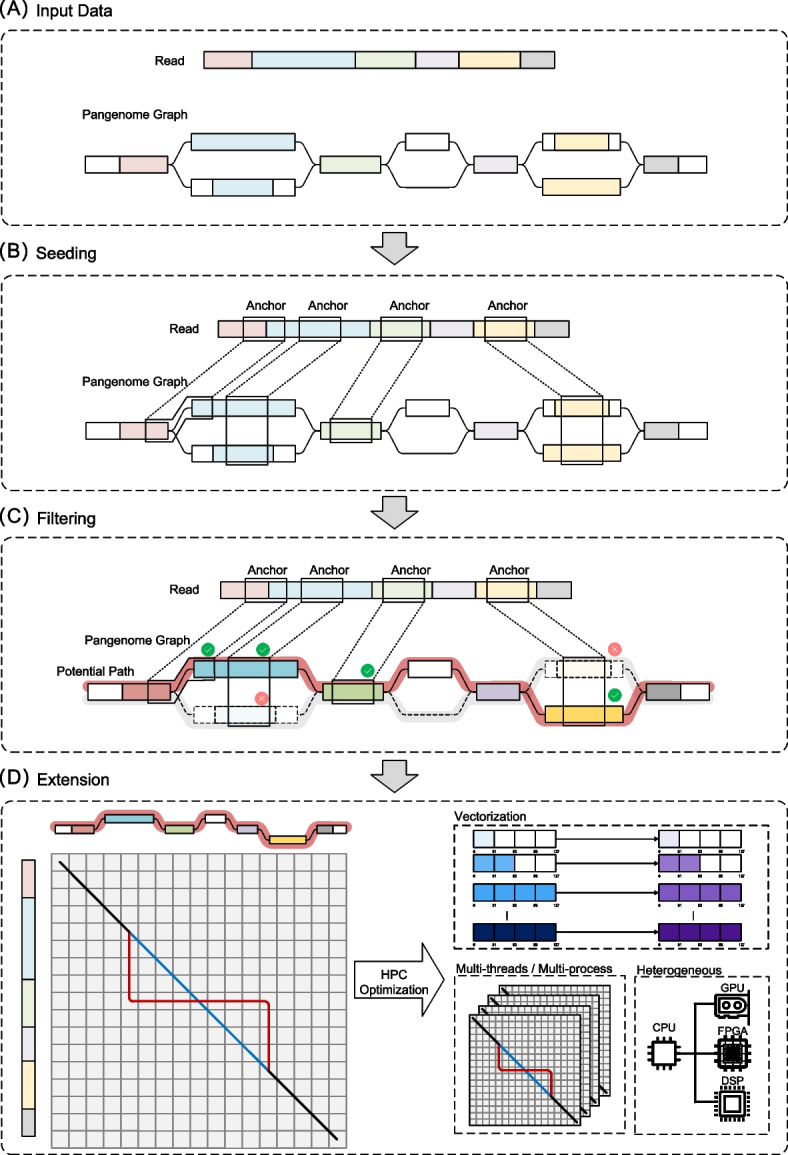
The workflow of “seed-and-extend” strategy in S2G mapping. **a** The inputs of S2G mapping: reads and a graph. **b** *Seeding* extracts seeds from reads and finds matches (anchors) between the graph and seeds. Seeds are usually indexed for fast query. **c** *Filtering* removes the unlikely matches in the candidate paths to reduce the computing costs afterwards. The checkmarks and crosses represent the selected anchor chains and discarded parts, respectively. **d** *Extension* performs base-level alignment between the candidate paths and the read for optimal alignment. The left side of the diagram illustrates the DP process in graph alignment, while the right side highlights high-performance computing optimizations that can be applied at this stage. The color scale in the vectorization section of the diagram illustrates the execution order of anti-diagonal computations in vectorized processing (to facilitate vectorization, the dynamic programming traversal is typically organized in an anti-diagonal order progression, ensuring mutually independent elements within each vector unit). Lighter shades correspond to anti-diagonals scheduled for earlier computation initiation

In S2G, aligning a string of length *M* to a graph of total text size *N* typically requires *O*(*NM*) time and space if using classical dynamic programming (DP) algorithms. Especially for large-scale data, it is impractical to directly apply these algorithms to exact alignment. Therefore, heuristic method is often used to reduce computation time, with most algorithms adopting the “seed-and-extend” strategy. This strategy mainly includes three steps: seeding, filtering, and extension, as shown in Fig. 2. In the seeding and filtering steps, the sequence is mapped to its likely positions. The subsequent extension step then completes the final precise alignment. It has been widely used in the mapping for both short reads and long reads [40, 45].

The first step of “seed-and-extend” strategy is *seeding* (Fig. 2b). Seeds are subsequences extracted from reads and graphs. Exact matching of seeds between reads and the graph helps locate the alignable regions in the graph. Therefore, seeding serves as a rough localization step, significantly reducing the search space from the entire graph to several regions. To improve the efficiency of seeds matching, it is necessary to index reads and graphs, and the query efficiency highly depends on the index used.

Matched seeds in the graph are called *anchors*. Due to the repetitive nature of genomes, many false positive anchors exist. The filtering step refines these anchors and further narrows the potential aligning regions. Currently, several techniques are used to filter anchors, mainly including *screening*, *clustering* and *chaining*. *Screening* involves removing anchors that do not meet specific requirements. *Clustering* groups anchors into clusters based on their similarity. *Chaining* selects and links an ordered subset of anchors that may form a possible aligning path.

**Fig. 3:**
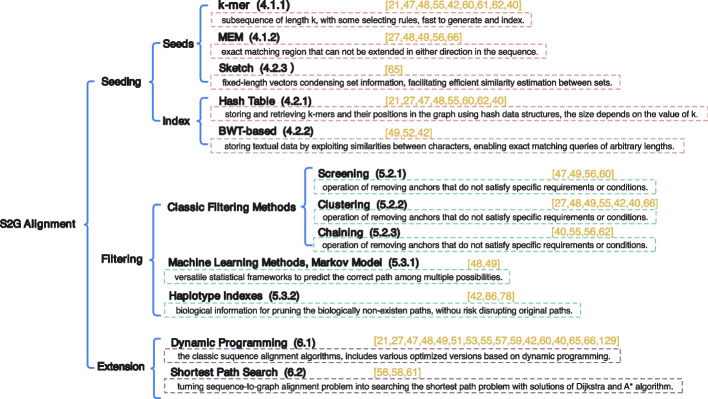
Methods used in each step of S2G alignment

**Fig. 4:**
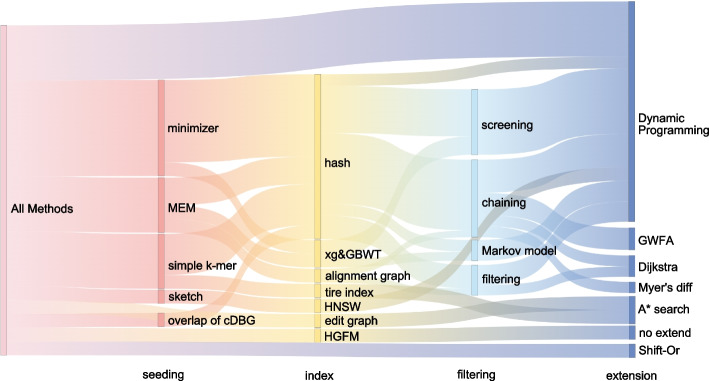
Flow distribution of S2G alignment algorithms across different alignment steps. In this Sankey diagram, each level represents different steps of S2G alignment, corresponding to the text at the bottom of each layer. The nodes within each level indicate the methods used in that step, and the width of the flows indicate the number of each method used across the steps

The *extension* step performs base-level alignment between the read and the potential regions to finalize alignment results. In S2G alignment, the potential regions may be a single path or a multi-branch path. Therefore, the extension needs to handle the complex graph topology, which requires more optimization. All the S2G alignment algorithms mentioned in this paper are listed in Table 1. Moreover, we summarized the methods used by these algorithms based on the three steps of alignment, shown in Figs. 3 and 4.

**Table 1:** Sequence-to-graph alignment algorithms

No.	Algorithm^a^	Year	Graph	File format (graph)	File format (alignmnet)	Read^b^
1	GenomeMapper [21]	2009	Sequence graph	.idx^g^/.mta^g^	.bed/.shore	Short
2	deBGA [27]	2016	DBG	index_route^g^	.sam	Short
3	BGREAT [46]	2016	DBG	.fa	.txt	Short
4	BrownieAligner [47]	2018	DBG	nodes.stage2^g^	.ncf	Short
5	VG map [48]	2018	VG	.vg/.gfa/.gbz	.gam/.bam/.gaf	Both
6	SOPANG [49]	2018	ED-string	.edz &.edzs^g^	.txt	Short
7	V-align [50]	2019	VG	.adj^g^	.txt	Short
8	HISAT2 [51]	2019	Sequence graph	genome^g^	.sam	Short
9	PaSGAL [52]	2019	VG	.vg/.txt	.txt	Both
10	Minigraph [53]	2020	Sequence graph	.gfa	.gaf	Long
11	GraphAligner [54]	2020	Sequence graph	.gfa	.gaf	Long
12	SPAligner [55]	2020	Sequence graph	-	-	Long
13	Vargas [56]	2020	Sequence graph	.gdef	.sam	Short
14	ASTARIX [57]	2020	Sequence graph	.gfa	.gaf	Short
15	HGA [58]	2021	Sequence graph	.txt^g^	.txt	Both
16	VG giraffe [41]	2021	VG	.vg/.gfa/.gbz	.gam/.bam/.gaf	Short
17	SeGram [59]	2022	Sequence graph	.gfa	.gaf	Both
18	ASTARIX2 [60]	2022	Sequence graph	.gfa	.gaf	Both
19	GraphChainer [61]	2023	VG	.gfa	.gam	Long
20	GWFA [62]	2022	Sequence graph	.gfa	.txt	Long
21	minichain [63]	2023	Sequence graph	.gfa	.gaf	Long
22	GED-MAP [39]	2023	ED-string	.gfa	.sam	Short
23	MG-SKETCH [64]	2023	DBG	.dbg	.gaf	Short
24	VG mpmap [65]	2023	VG	.vg/.gfa/.gbz	.gam/.bam/.gamp/.gaf	Short
25	PanAligner [31]	2024	Sequence graph	.gfa	.gaf	Song

No.	Seed	Index	Filtering^c^	Extension^d^	#Citation^e^
1	*k*-mer	Hash table	-	k-banded DP	281
2	Uni-MEM	Hash table	Clustering	Banded SW	87
3	Overlaps of cDBG	Hash table	Screening	DP	105
4	*k*-mer and MEM	Hash table	Clustering and MM	NW	30
5	SMEM	xg&GCSA2	Clustering and MM	SIMD SW	614
6	-	-	-	Shift-Or	26
7	-	-	-	SW	31
8	-	HGFM	-	-	8912
9	-	-	-	DP	44
10	Minimizer	Hash table	Chaining	SIMD SW	321
11	Minimizer	Hash table	Clustering and chaining	Bit-parallel NW	174
12	MEM	Alignment graph	Chaining	Dijkstra	18
13	-	-	-	DP	24
14	-	Edit graph	-	A-star search	32
15	-	-	-	DP	7
16	Minimizer	xg and GBWT	Clustering	SIMD X-drop DP	209
17	Minimizer	Hash table	Screening	Bitvector DP	44
18	*k*-mer	Trie index	-	A-star search	22
19	*k*-mer	Hash table	Chaining	Myer bitvector^f^	19
20	Minimizer	Hash table	Chaining	GWFA	7
21	Minimizer	Hash table	Chaining	GWFA	16
22	Minimizer	Hash table	Clustering and chaining	Semi-global SW	10
23	Sketch	HNSW	-	Semi-global SW	9
24	SMEM	xg and gcsa2	Clustering	SIMD banded DP	41
25	Minimizer	Hash table	Chaining	GWFA	18

^a^The algorithms contain aligners with and without the heuristic locating procedure

^b^The read types include short reads, long reads, and both of them

^c^Since many algorithms involve screening, which is relatively simple and common, it is possible that it is not explicitly mentioned in the corresponding papers. Therefore, in this column, “screening” refers to cases where the algorithm’s paper specifically indicates that only the screening method was used

^d^Both the SW and NW algorithms refer to the graph generalized version. The DP algorithm is ambiguous from the source reference

^e^The citation count provided here is based on the Google Scholar citations as of the time of manuscript submission

^f^GraphChainer first finds the chain of anchors by BFS and computes the edit distance using *edlib* [66]. It compares the output of the GraphAligner with its own output and returns the better alignment

^g^The formats marked with ^g^ refer to custom formats designed by the algorithm itself or intermediate results generated by the algorithm

## Seeding

In S2G mapping, the seeding step generates and matches seeds between reads and the graph, which plays an important role in locating potential mapping regions. It is also necessary to index seeds to improve query performance and reduce memory footprint.

### Generation of seeds

Selecting appropriate seeds is the primary challenge for mapping algorithms. The primary differences between various seeding algorithms lie in how these sequence fragments are selected and their length. The length of seeds significantly impacts the quality and efficiency of the mapping. Longer seeds will better capture unique sequence features, but with fewer anchors, which may result in an insufficient number of candidate regions even the miss of true mapping. Conversely, shorter seeds will produce a lot of anchors, but with many false hits, due to the repetitive nature of genomes. Moreover, more anchors will lead to higher computational burden.

Kristoffer Sahlin et al. [40] provide a comprehensive discussion on various types of seeds used in sequence-to-sequence (S2S) mapping. Most S2G algorithms, which are guided by S2S, utilize the extensively validated seeding methods. These methods for selecting seeds can generally be categorized into two types based on their strategies: static seeds and dynamic seeds.

#### Static seeds

A *k*-mer is a subsequence of length *k*. The length of a static seed is fixed, and different static seeding strategies select specific subsets of all *k*-mers as seeds according to predefined rules. GenomeMapper [21] is the first S2G aligner that utilizes *k*-mers as seeds. Algorithms such as BrownieAligner [47], ASTARIX2 [60], and GraphChainer [61] also use simple rules to select subsets of *k*-mers as seeds.

Currently, many S2G mapping algorithms select *k*-mers specifically based on the structure of graph. For instance, BGREAT [46] uses overlapping segments of the De Bruijn graph as *k*-mers, while GraphAligner [54] selects graph nodes as seed positions.

Minimizers, originating from S2S algorithms, have found extensive applications in S2G mapping. A minimizer is the *k*-mer with the minimum hash value in a sliding window of consecutive *k*-mers. The state-of-the-art algorithm minigraph [53] adopts minimizers as its seeds. VG giraffe [41] treats the graph as a set of haplotypes and selects minimizers from the sliding window on each haplotype. GraphAligner [54] selects minimizers per windows for each node in the graph and proves that the absence of seeds on branching paths does not affect the performance of long-read mapping. Additionally, there are other types of seeds. For example, Kristoffer Sahlin [67] developed a method to obtain fuzzy seeds with variable sizes, which allow one or more bases to be mismatched during the formation of anchor, rather than a perfect match. However, fuzzy seed has not yet been applied in pangenome graphs.

Some seeding methods, initially developed for traditional S2S alignment, show strong potential for extension to S2G alignment, such as spaced seeding [68], weighted minimizers [69], syncmers [70], strobemer [71], Genome-on-Diet seeds [72], and so on.

#### Dynamic seeds

Static seed entirely depends on the sequence, allowing the seeds to be stored and repeatedly used for different mapping targets. In contrast, dynamic seeds use different seeds for each query. Super Maximum Exact Matching (SMEM) [73] is one of the most representative for dynamic seeds. The acquisition of SMEM requires the generation of Maximum Exact Matching (MEM), which refers to the exact matching region that can not be extended in either direction. Obtaining MEMs in S2G involves extending all matched *k*-mer regions until exact match is no longer possible. The length of a MEM is variable but has a lower limit of *k*. SMEM is a special type of MEM that is not included in other MEMs, representing matches in the read that are not contained by longer matches [48]. Therefore, generating SMEM seeds is often slower than that of *k*-mer seeds due to the additional computation [40].

Heng Li later improved upon the Burrows-Wheeler Transform (BWT, a text compression algorithm as detailed in the “Text compression-based indexes” section) approach by introducing a reseeding method for overly long SMEMs [74]. This method involves reseeding using the longest exact matching sub-sequences within the SMEM that are sufficiently frequent. The approach then filters and refines these seeds to obtain MEMs of a specified length, enhancing the alignment process.

DeBGA finds longer matches using MEM within unitigs, which are called Uni-MEMs [27]. BrownieAligner [47] uses both *k*-mer and MEM seeds, where MEMs are shorter and specifically for reseeding in the non-match region. SpAligner used with SMEMs allows for easier and more dependable filtration of spurious matches [55].

### Storage and indexing of seeds

Most S2G mapping algorithms need to construct auxiliary data structures for graphs to facilitate fast queries. Different indexes may be suitable for different characteristics of seeds. The common indexing methods are implemented based on the following two algorithms: hash algorithm and text compression algorithm.

#### Hash-based indexes

Similar to S2S mapping, hash-based indexing is also widely used in S2G mapping problems. The hash-based index stores *k*-mers and their positions in the graph using hash data structures. Efficient indexing of *k*-mer seeds is achieved by employing a hash function to generate an integer value, typically 32- or 64-bit, which is then inserted into a hash table. In sequence alignment, specially designed perfect hash functions are used to establish a one-to-one mapping between *k*-mers and hash values. In practical applications, this approach typically ensures that *k*-mers in the hash table can be queried in constant time. However, this one-to-one mapping storage also results in significant memory overhead. Additionally, since hash values typically have a relatively fixed number of bits, they are more suitable for static seeds. However, there are also hash-based indexes designed for MEMs as well [47]. Due to the popularity of static seeds, hash indexing remains the primary solution.

#### Text compression-based indexes

From a computational perspective, S2G seeds are treated as strings. Numerous algorithms have borrowed and extended classic text processing algorithms, utilizing compression techniques to reduce the storage space required for seed indexing. Most of these mainstream indexing methods are based on the BWT. BWT index efficiently stores textual data by exploiting similarities between characters. It can enable exact matching queries of arbitrary lengths [75].

In the context of graphics, small variations can be represented by permuting textual characters, which allows the BWT-based index to be retained through linear mapping. BWBBLE introduces bubble and branch-like graphical structures using a BWT-based index with IUPAC encoding [76]. The textual characters permutation constrains encoding complex variations. Efforts for graphic generalization of BWT-based indexes were made.

Generalized Compressed Suffix Array (GCSA) [77] expands upon BWT, transforming multiple sequences into a prefix-sorted automaton. GCSA2 [78] enhances GCSA by employing a De Bruijn graph approximation strategy. Specifically, it constructs an index by transforming complex pangenome graphs into an equivalent De Bruijn graph representation with *k*-mer lengths up to 256 bp. Therefore, GCSA2 can maintain query accuracy while adapting to various types of graphs. VG map [48] and VG mpmap [65] employ GCSA2 to filter hits and cluster by distance on the graph, incurring time-space cost.

Additionally, since the number of *k*-mers in the read is less than in the graph, the query read can help reduce unnecessary costs. Positional Sensitivity Indexer (PSI) [79] efficiently indexes both reads and the pangenome graph simultaneously. It navigates the graph based on the reads, avoiding unnecessary retrieval of *k*-mers absent in the reads. This approach leverages the diploid nature of read queries, where each locus typically has only two alleles. By combining this concept with the traditional FM-index [80], PSI achieves highly optimized indexing performance, surpassing GCSA2 in index size, query time, and sensitivity.

The development of MEMs has been further applied to pangenomes. Gagie et al. [81] introduced the R-index, enabling faster localization of MEMs, although the R-index still has limitations for practical applications. MONI [82] represents the first comprehensive implementation utilizing the R-index, applying it to pangenomes to index and use multiple genomes as references. MONI constructs indexes with low memory usage and high speed, significantly enhancing the ability to identify MEMs across a large number of reference genomes.

#### Sketch-based indexes

Sketching is a technique first used in MHAP [83] for long-read overlap finding for assembly. It condenses a set of *k*-mers into fixed-length vectors, called sketches, by extracting representative features, which improves improving time efficiency compared to full-set pairwise comparisons. These sketches, comprising representative fingerprints, enable quick similarity estimation between sets of any size.

MG-SKETCH [64] introduced sketching into S2G mapping. It addresses seeding’s time-accuracy trade-off, employing Tensor Sketching (TS) and Hierarchical Navigable Small World (HNSW) index. TS converts sequences to tensor space for similarity estimation, while HNSW efficiently searches nearest neighbors in high-dimensional space using a hierarchical structure. It combines small-scale clustering networks and hierarchical indexing to enhance scalability on large datasets, distributing data points across levels for efficient neighbor search.

#### Optimizing methods of indexing

Both GCSA2 and hash-based index methods suffer from combinatorial explosion in complex regions with numerous variants, leading to substantial time and space consumption during mapping [79]. To mitigate this issue, heuristic approaches have been proposed, such as removing nodes with high in-degree or out-degree, eliminating small nodes, or excluding rare mutations [48, 51, 84]. However, these methods risk disrupting original haplotype paths and retaining biologically meaningless paths [85].

Due to the expensive nature of indexing the entire graph, many methods turn to indexing smaller but reasonable modules, such as haplotypes [41, 85]. For instance, VG giraffe [41] uses a BWT-based variant index known as GBWT [86], efficiently storing and querying per haplotype. GBWT is a multi-string FM index designed to index large collections of similar paths within a graph. It partitions the BWT into node-specific segments using local alphabets that encode successor nodes and terminal markers. Each segment employs run-length encoding for compacting storage and maintaining necessary operations. This design enables efficient indexing of pangenome graphs by preserving path topology and avoiding biologically invalid sequences.

## Filtering

This section introduces the methods of seed filtering or selection, especially those that will help the candidate mapping regions focus on one (or more) paths. This step is crucial because it narrows down the problem. Indeed, some methods can even transform the S2G mapping problem into S2S mapping problem.

Before filtering seeds, a clear coordinate system is necessary to determine the distance and positional information between seeds. Therefore, this section first introduces the construction of the coordinate system in graphs (the “Coordinate system building” section), then discusses some common classical seed filtering methods (the “Classic seed filtering methods” section), and finally presents new seed filtering techniques developed specifically for graphs (the “Specific filtering approaches for S2G mapping” section).

### Coordinate system building

A clear coordinate system can help further sequence mapping and downstream bioinformatics applications. Reference genome sequences offer a coordinate system for organizing genetic information, but when integrated into a graph, this clarity is lost [6]. This is because pangenomes, to address their large storage requirements, fold the graph, resulting in more complex structures such as cycles [13]. Besides, many filtering methods rely on computing distances between seeds, which can be challenging due to the topology of graph [54]. To address the problems above, a well-defined local or global coordinate system is essential [6]. This coordinate system should ensure the uniqueness of each node, which may involve sorting the nodes or making the graph sortable.

The early simple way of building a global coordinate is integrating VCF with a linear reference, supported by many algorithms. This method can directly use the reference position as coordinate. However, the reference may not cover the entire graph [6]. Also, it does not incorporate insertions into the coordinate system, posing limitations in encoding long insertions [53].

These constraints have spurred the advancement of comprehensive coordinate frameworks for genome graphs. minigraph [53] adopts a stable coordinate system of encoding names and coordinate offsets for all sequences used in graph construction. It retains more linear characteristics by prohibiting the collapse of distinct regions from the same sequence, which may lead to a larger but simple graph.

Other methods directly utilize the topology of the graph to construct the system. They first detect *superbubbles* (a subset of snarls [48, 87]), which are induced acyclic subgraphs with one unique entrance node, one unique exit node, and some amount, possibly zero, of internal nodes [54, 88]. Different methods for processing superbubbles exist. In study of Paten, et al. [87], the graph topology was decomposed to hierarchically describe the graph. This decomposition has been rigorously validated through mathematical proof and has already been applied in the field of sequence alignment [26].

Visiting all nodes of the chain of superbubbles in order can be a simple local coordinate system construction method. GraphAigner uses a breadth-first search (BFS) algorithm for position assignment. Also, we can obtain local ordering by topological sorting of a DAG, which arranges the nodes according to their dependencies and is widely used in chaining algorithms for minigraph [53], GraphChainer [61], and other methods [89].

Current alignment tools natively employ different coordinate systems, most of which fall into the categories described earlier. These coordinate systems, however, are interconvertible, allowing them to be adapted for use across various tools. Despite this flexibility, the use of different coordinate systems can still introduce challenges in seed generation, filtering during alignment, and downstream bioinformatics analyses following sequence alignment.

### Classic seed filtering methods

#### Screening

To distinguish from the chapter title, we use screening here to refer to filtering out certain anchors based on specific criteria. Screening is the most straightforward and easy-to-use method for seed filtering. The basic idea is to set thresholds based on empirical information and remove seeds according to these thresholds.

Common criteria for deletion include factors, such as being too short [46] or overlapping with others [55]. Additionally, seeds with high hit counts are often given priority for removal [48], which would result in mapping failure in long repetitive regions [63].

Many studies have designed more sophisticated filtering criteria. For example, the following approaches go beyond simple exclusion. GASSST [90] uses a precomputed table to assist in filtering, while rHAT [91] retrieves windows containing anchors through an RHT and retains the top *M* windows, where *M* is a user-defined parameter.

#### Clustering

Clustering is commonly used to group seeds that are close in distance together. It typically selects the seeds with distance under a threshold as a cluster, and then scores the seed hits by criteria such as cluster size or uniqueness. Clustering seeds allows computations to focus more on seed-dense areas, reducing the impact of scattered sparse seeds and improving mapping performance. Some algorithms align reads directly around clusters [27, 41]. The clustering algorithm in VG mpmap [65] defines spatial proximity metrics for seed sequences and employs dynamic programming to group seeds into clusters, prioritizing those achieving the highest alignment scores as the final selected clusters. Typically, GraphAligner [54] employs the clustering algorithm from minimap [92] with brief refits.

Nevertheless, this method can lead to inaccuracies in locating the correct positions within graphs for connecting a single seed or cluster along a long read [61]. When seeds are clustered in multiple regions of the graph and separated by distance, it becomes challenging to extend from one seed through erroneous regions to reach the next accurate region, resulting in short mapping covering different parts of the read instead of a long mapping of the entire read. Moreover, a seed may generate multiple false hits in the graph, hindering the accuracy and effectiveness of clustering. Additionally, clustering may overlook long distances between seeds, potentially affecting the mapping result, especially when some of these gaps are inherent in genome.

#### Chaining

##### Co-linear chaining algorithm in S2G mapping 

Co-linear chaining algorithm, a mathematically rigorous approach for finding promising anchor chains, can address those issues present in the aforementioned methods. It is well studied in S2S mapping [93–95], and widely applied in modern long read S2S aligners [96–98], with the most popular one being minimap2 [99]. This dynamic programming chaining algorithm outputs a chain of anchors with maximum score. A chain is a subset of anchors maintaining the same order of intervals in both the sequence and the reference. In these algorithms, the gap cost function is generally defined as the sum of the weights of the anchors in a chain minus the penalty for gaps between anchors. However, the scoring schemes differ significantly, often depending on the coordinate system used. For example, in Minichain [63], the gap penalty represents the length of the shortest path between adjacent anchors, while Ghanshyam et al. [100] incorporate haplotype-switching penalties. The specific settings for anchor weights and gap penalties greatly influence the ability to identify an optimal anchor chain.

##### Improvement of co-linear chaining

The efforts of generalizing this solution to graphs have been made. minigraph utilizes a similar chaining algorithm of minimap2 but with different scoring criteria and an added heuristic to improve speed [53]. SPAligner [55] implements a co-linear algorithm of dynamic programming, assigning seed weights based on their span and defining compatibility between anchors based on minimum distances in the graph versus distances in the read. However, the time complexity of checking compatibility, calculated by distances, is quadratic [61].

Some recent studies have adopted concepts and algorithms based on minimum path cover (MPC) to address the co-linear chaining problem in sequence-to-graph alignment. MPC is the smallest set of paths in a DAG such that every node in the graph is included in at least one path. The co-linear problem between a sequence and a DAG remained unresolved until a sparse dynamic programming framework for MPC problem was proposed by Makinen et al. [89]. This study solves it by mimicking co-linear chaining algorithms between sequences on each path of the minimum path cover, resulting in the algorithm generated by this framework being only slower on sequences by a factor of *k* compared to the corresponding algorithm. The co-linear problem restricted to DAGs, after a preprocessing step taking $O(k(|V|+|E|)log(|V|))$ time, can be solved in $O(kNlog(N)+k|V|)$ time when overlapping between suffixes and prefixes of anchor paths is prohibited. Here, |*E*| and |*V*| are the number of edges and nodes in the graph, respectively. *N* is the number of input anchors, and *k* is the width of the directed acyclic graph, defined as the minimum number of paths required to cover each node in the graph at least once. Further research has experimentally shown that the average width of the human variation graph is typically small [101], resulting in a lower time complexity for the aforementioned dynamic programming algorithm compared to others.

Inspired by the previous sparse DP framework, many advancements have been made. GraphChainer [61] proposed an extension version allowing one-node suffix-prefix overlaps between anchor paths. It means that the anchor can lie on a sequence that connects two nodes which are mutually preceding and succeeding. This approach enables the construction of more suitable chains in certain datasets, compared to methods that do not allow such span. The algorithm’s running time is divided into preprocessing the graph and solving co-linear chaining, with the latter taking *O*(*Nlog*(*N*)) time for constant-width graphs. Allowing one-node suffix-prefix overlaps expands the solution space and potentially enhances the final solution. Minichain [63] designs the gap cost function that fits the sparse DP framewok above [89] and solves the chaining problem optimally in *O*(*kNlog*(*kN*)) time. It has been applied to human pangenome DAGs built by using 94 haplotypes [9]. Building upon minichain, the research group embarked on new investigations, employing different penalty functions to explore deeper into the co-linear chaining problem between sequences and graphs. Ghanshyam et al. [100] introduce a haplotype-aware co-linear chaining algorithm to reduce the spurious read mapping to those paths that are unlikely recombinations of the known haplotypes. It uses recombination penalty for a haplotype switch to reduce the probability of formation of chains that switch between different haplotype paths. The problem can be solved in *O*(|*H*|*Nlog*(|*H*|*N*)) time, assuming that a one-time preprocessing of the DAG is performed in *O*(|*E*||*H*|) time, where *H* is the set of haplotypes represented in the graph. PanAligner [31] introduces a gap penalty function that considers the scenario of calculating gaps for cycles to address the problem of co-linear chaining between sequences and graphs with cycles. The algorithm first employs a heuristic method of removing back edges of strongly connected components to obtain a DAG that can approximate the optimal MPC. Then, the algorithm iterates to continually refine the solution. This algorithm solves the chaining problem on cyclic graphs in $O(\Gamma _c|P|N log(N) +|P|N log(|P|N) )$ time after a one-time preprocessing of the graph in $O((\Gamma _l + \Gamma _d + log(|V |))|P||E|)$ time, where $\Gamma _c$, $\Gamma _l$, and $\Gamma _d$ are the parameters of iteration counts and *P* is the number of paths in the graph.

Practically, the selection of chaining algorithm usually accounts for read feature, graph structure, etc. Recent methodological developments explore distinct theoretical approaches: Minichain [63] and GraphChainer [61] have developed specialized chaining strategies tailored to the characteristics of long reads. Furthermore, both PanAligner [31] and minichain [63] have implemented topology-aware designs in their chaining algorithms to accommodate specific graph architectures. These methodological innovations demonstrate the potential theoretical advantages across diverse pangenomes and may provide conceptual foundations for novel algorithmic developments.

#### Approaches for accelerating filtering

Effective filtering algorithms can significantly reduce the overall computational load and improve alignment accuracy. Additionally, filtering speed itself is a critical consideration, and numerous studies leverage high-performance computing (HPC) theories and algorithms to enhance the efficiency of this process. For instance, tools like GRIM-Filter [102], Shouji [103], and SneakySnake [104] focus on optimizing the use of various hardware platforms, designing more efficient memory access patterns, and implementing parallelization to accelerate filtering. These approaches illustrate how hardware-aware optimization and HPC strategies can contribute to faster and more accurate filtering in sequence alignment.

### Specific filtering approaches for S2G mapping

Restricting mapping to the most probable paths is an effective advantage strategy in S2G mapping. This approach reduces computational costs and decreases the likelihood of mapping sequences to paths that have not been observed in natural populations. Some new novel methods have been proposed.

#### Markov model

Rather than rigorous mathematical computational methods for finding paths, methods of using machine learning model to predict the most likely paths are alternative, especially in DBG [47, 48]. Markov models (MMs) are versatile statistical frameworks used in various domains [105]. In graph mapping tasks, the aligner leverages information from previously visited nodes and other reads to predict the correct path among multiple possibilities, effectively avoiding mapping against false paths. MM provides valuable information that is absent in the De Bruijn graph for improving the accuracy.

#### Haplotype-based indexes

In addition to artificially constrained mapping paths, naturally occurring biologically meaningful paths are preferable choices. Graph Burrows-Wheeler Transform (GBWT) is an index built upon the haplotypes can utilize haplotype information to expand paths limited to haplotypes [86]. The other method, proposed by Mokveld and Linthorst [85], decomposes the graph into haplotype-mappable sequences, facilitating the use of standard aligners for matching without requiring graph pruning or filtering. This method is limited to short read mapping.

## Extension

The extension step aligns the remaining query read seeds with the graph to find the best possible alignment. However, the challenge arises due to the complex topological structures of pangenome graphs. For example, the positions of multiple alleles may result in numerous branches, and repetitive regions can lead to a large number of cycles and nested cycles. Extending seeds across these branches structures becomes particularly difficult. Some algorithms based on S2S alignment primarily use DP and shortest path search techniques to design solutions and address the aforementioned issues.

### Dynamic programming

In traditional sequence alignment, base-level sequence alignment is primarily performed using the DP algorithms [42, 106]. If the lengths of the query and reference sequences are *n* and *m*, the time complexity of traditional approaches based on Needleman-Wunsch (NW) [106] and Smith-Waterman (SW) [42, 107] algorithms is *O*(*nm*). Navarro et al. [108] extended the NW algorithm to graph structures, and Lee et al. [109] extended the SW algorithm to directed acyclic graphs, which called Patial Order Alignment (POA). Both these approaches use DP matrices for S2G alignment, which requires significant memory storage and lengthy iterative backtracking [110].

#### Accelerating S2G with parallel computing

Common parallel acceleration techniques include utilizing SIMD instructions, multiprocessing programming, heterogeneous computing and so on [107, 111]. These methods also can be categorized into inter-sequence parallelism and intra-sequence parallelism.

PaSGAL is a inter-sequence parallel alignment algorithm that utilizes SIMD instructions. It employs a three-phase parallel approach, leveraging task-level parallelism for computing base-level alignments [52]. Compared to VG without heuristic implementation, PaSGAL achieves a significant speedup, reducing running time by several-fold.

Vargas, a heuristic-free algorithm, aims to find optimal alignments [56]. It utilizes multiprocessing programming and SIMD instructions for acceleration, reaching a peak speed of 456 billion cell updates per second.

In 2021, Feng and Luo introduced HGA, an optimized aligner designed for heterogeneous processors [58]. HGA employs CPU-GPU cooperative processing and enhances memory locality, outperforming PaSGAL and other aligners.

The first tool to truly utilize high-performance methods for accelerating sequence-to-graph alignment is SeGraM [59]. SeGraM combines high-bandwidth memory to leverage low-latency and highly parallel memory access, effectively eliminating memory bottlenecks. As a result, it achieves a significant acceleration ratio.

Scoorge [112] improves upon GenASM [113] by reducing data movement, memory usage, and the number of operations within the GenASM algorithm. It offers implementations for CPU, GPU, and ASIC platforms, creating a fast alignment tool suitable for various hardware configurations. The customized hardware version significantly reduces energy consumption and chip area, providing a novel approach for efficient sequence alignment.

Recently, NVIDIA developed a GPU version of VG giraffe by leveraging heterogeneous computing approach, enhancing its performance and efficiency [114]. This work is integrated into NVIVIA’s Clara Parabricks package, a software suite designed for accelerated analysis in genomics.

These advancements in parallelization and optimization techniques have significantly improved the speed and efficiency of base-level sequence alignment, offering better solutions for various alignment tasks compared to traditional DP-based methods.

#### Optimization for the loops in graph

Recent advancements in sequence alignment algorithms have improved efficiency. However, addressing the loops of graph remains a significant challenge due to the inherent properties of POA [6]. Loops of graph, where a node $n_i$ is reachable from itself, are common in genomic data.

Existing approaches address loops of graph by preprocessing them to ensure alignment correctness. VG map [48] employs an “unrolling” method to transform cyclic graphs into acyclic ones, while VG giraffe [41] leverages haplotype-aware strategies to avoid cycles. However, this approach may encounter exponential space and time issues in extreme cases [53]. V-Align identifies feedback vertex sets during preprocessing to enable POA on cyclic graphs [50], with time complexity depending on the set’s size. Rautiainen et al. extended Myers’ bitvector algorithm for semi-global alignment to handle S2G alignment with cyclic graphs [115]. Despite bit-parallel optimizations, the high cost limits their practicality as graph scale increases.

#### Optimization for the multipaths in graph

VG mpmap [65] extends conventional dynamic programming algorithms to enable multipath alignment, where sequence is aligned to multiple paths rather than a single path in a graph. This extension allows the algorithm to handle alignment uncertainties at known variants or splice junctions by maintaining a graph of possible alignments. This method not only enhances the accuracy of alignments but also preserves the topological complexity of a pangenome graph, enabling downstream applications to utilize alignment uncertainties effectively.

#### Optimization for similar sequences

Santiago Marco-Sola et al. [43] introduced Myers’s difference algorithm [116] into S2S alignment, and proposed the Wavefront Alignment (WFA) algorithm, which replaces the edit distance with the gap-affine model. In contrast to classical methods, WFA methods do dynamic programming over scores rather than performing dynamic programming over the sequences. The algorithm has a time and space complexity of *O*(*ND*), where *N* is the sum of the lengths of the two sequences, and *S* is the minimum penalty. The WFA algorithm has garnered significant attention due to its efficiency in both memory and time usage [44, 117].

Expanding WFA to S2G alignment, the Graph Wavefront Alignment Algorithm (GWFA) was developed for aligning sequences to graphs [62]. GWFA stands out for its improved speed, particularly for closely matched sequences, with its runtime only moderately increasing with the edit distance of the optimal alignment. Compared to other exact S2G alignment algorithms, GWFA demonstrates up to a four orders of magnitude speed improvement on real datasets. Additionally, GWFA incorporates a graph pruning heuristic, further enhancing its efficiency, particularly on large graphs. Currently, some algorithms integrate GWFA as their alignment module [31, 63].

### Shortest path search algorithm

DP algorithms in S2G alignment algorithms often suffer from poor alignment efficiency, especially when the average running time approaches the worst-case complexity [57]. To address this issue, Pesho Ivanov et al. [57] introduced ASTARIX, which utilizes A* algorithm, a widely used heuristic search algorithm in graph theory, to search the shortest path. By treating S2G optimal matching as a shortest path problem, ASTARIX incorporates a heuristic function and various optimization strategies to ensure accuracy while significantly reducing computational time. In short-read scenarios, ASTARIX outperforms PaSGAL by returning optimal alignments one to two orders of magnitude faster and supports reference graphs with cycles.

To further enhance alignment efficiency and accommodate graphs of varying lengths, ASTARIX2 leverages a seed-based heuristic approach [60]. This method segments reads into seeds, searches for seeds within the graph, and guides the A* algorithm to explore paths efficiently. By combining this heuristic with the shortest path search algorithm, ASTARIX2 achieves a minimum 60$\times$ speedup compared to other DP-based aligners. However, the seed-based heuristic increases memory consumption due to the necessity of maintaining the search queue, limiting its applicability in larger graphs.

## Other methods

### Non-seed-and-extend mapping

There also exist seed-independent S2G mapping algorithms, with HISAT2 [51] being a notable example. Kim et al. employed an index based on the BWT and FM-index [80] in the RNA alignment algorithm HISAT [118]. It utilizes a global index anchor matching and multiple small local indexes for rapid alignment, which are later adopted in S2G aligner HISAT2 [51]. They further extend this index to graphs using a prefix-sorted automaton, and developed graph FM and hierarchical graph FM. Since reference genomes are typically large, global indexing is not cache-friendly. HISAT2 adjusts small local sequences to fit cache size to increase cache hits. Thus, this algorithm may be more suitable for small-scale variations.

### On-line alignment

The core of sequence alignment is approximate pattern matching, which can be performed in offline or online modes. Offline mode means preprocessing the entire text beforehand to construct an index, which is then used for efficient matching. The approaches outlined in the earlier sections of this review can largely be categorized as offline alignment algorithms. In contrast, online mode refers to scanning the text sequentially from start to end without preprocess, directly searching for the pattern during the scan [119, 120]. The KMP algorithm [121] is a classic online matching algorithm, which finds a pattern in a text efficiently by pre-processing the pattern to build a partial match table used to skip characters and resume search upon mismatch.

The core advantage of online alignment is its low resource consumption, making it faster in some scenarios [39, 120]. Since online methods do not require additional preprocess steps, they achieve higher efficiency for single queries against a genome compared to offline methods. Moreover, online alignment could process streaming data in real time without the need to acquire the complete dataset. However, once the preprocessing step (typically indexing in genomic sequence alignment) is completed, offline methods generally achieve faster extension speed than online methods.

Inspired by some online string matching algorithms [120, 122], existing online S2G mapping algorithms also adopt ED-strings [39, 49]. Iliopoulos et al. proposed a solution with a time complexity of O($\alpha$ $\gamma$mn) and a space complexity of *O*(*N*) for the Elastic-Degenerate String Matching (EDSM) problem [123]. Here, $\alpha$ represents the maximum number of strings in any segment of the text, $\gamma$ denotes the maximum number of segments spanned by any occurrence of the pattern, *m* represents the length of the pattern, and *n* represents the length of the text. Grossi et al. introduced an algorithm that constructs a suffix tree for the pattern and scans the target string online [38]. It remembers pattern prefixes occurring as suffixes in the target string and extends partial matches. The algorithm verifies complete pattern occurrences in the target string, efficiently searching for matches and complete patterns. SOPanG [49], an online text searching algorithm over a pangenome, implements this method with $O(N\lceil m/w \rceil )$ time complexity, where *m* is the length of pattern, *N* is the size of text, and *w* is the machine word size (in bits). It utilizes the Shift-Or bit-parallel technique [124] and supports approximate string matching with k-mismatches, achieving practical speed one order of magnitude faster than theoretical estimates.

## Future directions

S2G mapping is a cornerstone in leveraging pangenomes for genomic analyses. Despite its transformative potential, S2G mapping faces some challenges that hinder its widespread adoption and practical application. These challenges span diverse aspects, from algorithmic design and data standardization to coordinate systems and computational efficiency. In this section, we discuss the main obstacles in S2G mapping and highlight opportunities for future research.

S2G mapping algorithms face diverse issues caused by graph structure. Some algorithms can only work with DAGs and struggle to handle complex cyclic or branching regions within the graph. These regions, often repetitive sequences involved in regulatory functions or hotspots of individual genetic variation, are particularly difficult to map accurately. To address this, many approaches simplify such regions to ease the mapping process [16, 54]. However, this simplification often compromises alignment accuracy in these regions. On the other hand, preserving the complexity of these regions can disrupt the mapping in other parts of the graph, ultimately reducing both overall mapping quality and computational efficiency. Developing innovative mapping methods that strike a balance—enabling precise alignment of complex regions while maintaining high accuracy and efficiency across the entire graph—remains a critical and active area of research.

Another challenge in S2G mapping is the standardization of file formats. Unified formats for graph representation and alignment outputs are critical for compatibility between tools and facilitation of downstream analyses. As summarized by Table 1, significant variability existed in the file formats for graph representation and alignment outputs before Heng Li introduced the GFA and GAF formats [53]. However, a consensus on standard file format has yet to reach. For graph representation, the GFA-spec [125], now advanced to GFA v2.0, shows promise as a potential standard. Nevertheless, this situation also exists in the standard format of graph alignment results. Alignment results are increasingly adopting the GAF format, with complementary tools like gfatools [126] emerging for efficient processing. Moreover, to integrate with existing bioinformatics tools designed for linear reference genomes, it is often necessary to convert graph alignment results into linear alignment results, such as SAM format. The accuracy implications of this conversion remain underexplored. The lack of standardized formats creates additional barriers, as many analysis tools support only specific formats, significantly complicating the adoption of pangenomes in bioinformatics workflows.

The establishment of an appropriate coordinate system is also a challenge for S2G mapping. Linear reference sequence mapping tools typically rely on a common reference, such as GRCh(in various versions). This standardization facilitates the reuse and analysis of experimental results and datasets across studies. In contrast, pangenome graphs lack such a universal coordinate system, leading to discrepancies that hinder the interoperability of experimental data and complicate downstream analyses. Moreover, variations in how distances are defined within the graph can affect essential stages, such as anchor filtering. Although methods for converting between coordinate systems exist, some fundamental issues remain unresolved. These include the constitution of an optimal coordinate system, the impact of different coordinate systems on algorithm performance, and fair comparisons across algorithms operating under disparate coordinate system. Addressing these challenges would facilitate a more seamless integration of pangenomes and S2G mapping with existing bioinformatics data analysis workflows.

S2G mapping, compared to linear reference mapping, involves significantly more complicated input data, raising concerns about mapping speed. Some researchers use HPC techniques to improve mapping efficiency [102–104, 112], such as vectorization, multi-thread, multi-process, and heterogeneous computing. However, these optimizations also present several challenges. The major one is that many methods are designed for specific hardware, requiring substantial redesign for a new platform. In the context of HPC for mapping algorithms, improving method portability and simplifying usage are important areas for future research.

In summary, overcoming the challenges in S2G mapping will require interdisciplinary efforts, combining algorithmic innovation, standardized practices, and improved computational frameworks. Achieving a balance between accuracy, efficiency and usability is critical for the effective integration of S2G mapping into mainstream bioinformatics workflows. Addressing these issues not only enhances the practicality of pangenome-based analyses but also paves the way for broader adoption and advancements in genomics research.

## Data Availability

No datasets were generated or analyzed during the current study.
